# Heading back into the perfect storm: increasing risks for disease emergence in Brazil?

**DOI:** 10.1590/0037-8682-0640-2021

**Published:** 2022-06-06

**Authors:** Sérvio Pontes Ribeiro, Mariana Moncassin Vale, José Alexandre Felizola Diniz-Filho, Geraldo Wilson Fernandes, Alexandre Barbosa Reis, Carlos Eduardo de Viveiros Grelle

**Affiliations:** 1 Universidade Federal de Ouro Preto, Núcleo de Pesquisas em Ciências Biológicas, Ouro Preto, MG, Brasil.; 2 Universidade Federal de Ouro Preto, Departamento de Biodiversidade, Evolução e Meio Ambiente, Laboratório de Ecologia do Adoecimento e Florestas, Ouro Preto, MG, Brasil.; 3 Universidade Federal de Minas Gerais, Departamento de Parasitologia, Laboratório de Fisiologia de Insetos Hematófagos, Belo Horizonte, MG, Brasil.; 4 INCT em Ecologia, Evolução e Conservação da Biodiversidade, Goiânia, GO, Brasil.; 5 Universidade Federal de Rio de Janeiro, Departamento de Ecologia, Rio de Janeiro, RJ, Brasil.; 6 Universidade Federal de Goiás, Instituto de Ciências Biológicas, Departamento de Ecologia, Goiânia, GO, Brasil.; 7 Universidade Federal de Minas Gerais, Departamento de Genética, Ecologia e Evolução, Laboratório de Ecologia Evolutiva e Biodiversidade, Belo Horizonte, MG, Brasil.; 8 Universidade Federal de Ouro Preto, Departamento de Análises Clínicas, Laboratório de Imunopatologia, Ouro Preto, MG, Brasil.

Brazil has a recent history in health sciences, particularly parasitology and tropical medicine, with inspirational names such as Carlos Chagas and Oswaldo Cruz from the early XX century^1^. However, it took another century for Brazil to reach what could be called a science-driven society. After decades of insufficient jobs in science^2^, from 2002 to 2015, the country continuously expanded its positions for scientists, increasing the number of public universities, graduate programs, and investments in science and technology (https://geocapes.capes.gov.br/geocapes/). As a result, a scientifically and technologically sound Brazil emerged along with increased human development index (HDI), food, health, and environmental security^3^. Brazil’s unified health system (SUS, in Portuguese), the largest and most comprehensive globally, opened up access to epidemiologic data and adopted the “One Health” paradigm^4^. Important initiatives have brought about a solid partnership between health services and science, especially in neglected tropical diseases. An important example is the establishment of the University of São Paulo’s “Nucleo de Medicina Tropical” in Pará State, a center of excellence in tropical medicine in the heart of the Amazon, the largest tropical forest in the world^5^. Over time, the sanitarian-driven public health approach has become environmentally driven, and the urgency of reconciliation between health and nature conservation sciences has become obvious^6^. 

In the late 1990s, deforestation in the Amazon skyrocketed due to the national and international demand for commodities such as soy and beef^7^. However, in the first 16 years of the XXI century, deforestation and likelihood of zoonotic disease spillover reduced without sacrificing the economy. From 2004 to 2012, the Brazilian Action Plan for the Prevention and Control of Deforestation in the Amazon (PPCDA in Portuguese) reduced deforestation by approximately 80%, while the gross domestic product (GDP) in the Amazon increased by 141%^7^ ^,^ ^8^. Unfortunately, the lack of quality data before the 1990s prevented adequate investigation of neglected tropical diseases (NTD) epidemiologic trends, as demographic and compulsory data on NTD were hardly available. However, from 1990 to 2016, evidence-informed, efficient, and affordable interventions^9^ effectively reduced NTD daily rates by 45% in the whole country, although further research is required.

Science-driven policies and data transparency came after decades of irresponsible exploitation of natural resources. Following a long period as a country dedicated to agriculture and post-colonial exploitation, in the 1950s, the political willingness to modernize the country came with unregulated development, which left the most populated regions exposed to pollution and deforestation.

The intensity and extension of land degradation until the 1980s, mainly in Southeast Brazil, military Amazon occupation projects, and an increasing urban population with poor living conditions, were heading Brazil towards a perfect storm of sanitary and ecological crisis. Then, in the 80s, things started to change, with the new 1988 constitution firming strong environmental laws and the National Institute of Spatial Research’s provision of accessible deforestation data (http://terrabrasilis.dpi.inpe.br/en/home-page)^10^. Nevertheless, the present administration elected in 2018 interrupted this new and virtuous path. As a result, science-driven health and environmental policies started to be undermined, while the scientific community started to face difficulties accessing public data due to new rules that constrain access (a reality reflected in the 2 years delay in the country’s demographic census). 

**FIGURE 1:**
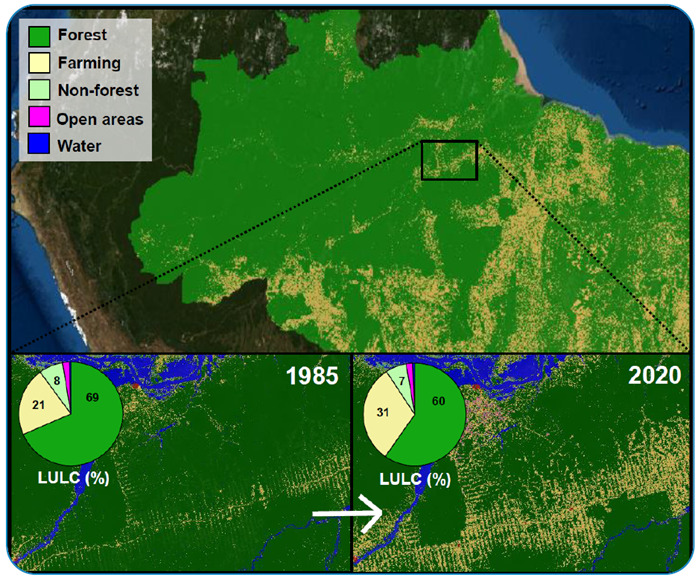
Amazon deforestation through time. Northern Brazilian Amazon in 2020 (top) showing deforested areas. The details show deforestation scares in 1985 (left) and 2020 (right) in the territory between the cities of Santarém and Altamira in Pará State. The graph presents land-use land-cover cover (LULC) in 2020 for the entire Brazilian Amazon, showing the LULC percentages. Sources: "Projeto MapBiomas - v 6.0, from the annual series of maps of Brazilian land cover and use da Série Anual de Mapas de Uso, accessed on 10 January 2022, https://plataforma.brasil.mapbiomas.org/"

The trend of increasing deforestation began in 2016 and has accelerated dramatically since 2019^7^. In addition to increasing the risks of new spillover of zoonotic diseases^8^, ongoing deforestation leaves a trail of re-emergent NTDs. Since 2017, economic activities related to deforestation in the Amazon have increased the incidence of 11 NTDs and diseases directly related to environmental degradation, such as water-borne diseases, reverting a trend, and preventing the eradication of these diseases, all related to poverty^11^. Eventually, this throwback in health and environmental protection warns us of a worsening scenario (Figure 1). These figures are early warnings of the wrong directions the country is taking, heading back to a perfect environmental and health storm, with increasing risks of new emergent diseases, including potentially pandemic ones.

Particularly for the Amazon, the present detachment between science and policymakers has driven re-emergent diseases and opened up a large road for coronavirus disease 2019 (COVID-19) to reach indigenous communities^11^. The vulnerability of Manaus International Airport and the risk of the city becoming a powerful spreader of the disease were predicted before the full dissemination of COVID-19 in the country^12^. The city’s natural susceptibility to emergent outbreaks of respiratory diseases was reviewed by comparing the effects of the present pandemic on its population with a similar humanitarian catastrophe during the Spanish flu. In both cases, the excessive death toll was strongly driven by poor political and social decisions^12^. History shows that poor leadership wiped out populations during pandemics and might be a stronger driver of pandemic death rates than any other evolutionary following up^8^. Worryingly, the environmental and public health scenario is deteriorating all over the country, not just in the Amazon. 

Currently, Brazil has an old, long-term environmental degradation in its Southern territories, whereas the Northern and Western territories are new deforestation frontiers. As a result, forest remnants previously protected by law enforcement are increasingly threatened by illegal exploitation. Additionally, the whole country has been under the pressure of increasing urban populations and since 2017, witnessing poverty coming back and an increasing lack of governance. Hence, a perfect storm to trigger emergent and re-emergent diseases with high pandemic potential is brewing again. 

Brazil has the resources to monitor and prevent the resurgence of any tropical diseases and a future pandemic in the country; however, it is necessary to resume investments and respect to scientific institutions. Moreover, there is an urgent need to strengthen dialogue and collaboration between organizations and scientific societies devoted to public health and biodiversity conservation. 
